# Umbilical cord blood versus unrelated donor transplantation in adults with primary refractory or relapsed acute myeloid leukemia: a report from Eurocord, the Acute Leukemia Working Party and the Cord Blood Committee of the Cellular Therapy and Immunobiology Working Party of the EBMT

**DOI:** 10.1038/s41408-019-0204-x

**Published:** 2019-04-12

**Authors:** Frédéric Baron, Myriam Labopin, Annalisa Ruggeri, Gerhard Ehninger, Fransesca Bonifazi, Matthias Stelljes, Jaime Sanz, Gernot Stuhler, Alberto Bosi, Nicolaus Kröger, Maria Teresa Van Lint, Arnold Ganser, Edouard Forcade, Mohamad Mohty, Eliane Gluckman, Arnon Nagler

**Affiliations:** 1 GIGA and CHU of Liege, University of Liege, Liege, Belgium; 2 Department of Haematology, Saint Antoine Hospital, Paris, France; 3 EBMT Paris Study Office/CEREST-TC, Paris, France; 4 INSERM UMR 938, Paris, France; 5 Sorbonne University, Paris, France; 6 Eurocord, Saint Louis Hospital, Paris, France; 7 Dipartimento di Oncoematologia e terapie cellulari, Ospedale Pediatrico Bambino Gesù, Roma, Italy; 8 Universitaetsklinikum Dresden, Medizinische Klinik und Poliklinik I, Dresden, Germany; 9 S.Orsola-Malpighi Hospital, Institute of Hematology and Medical Oncology, Bologna University, Bologna, Italy; 10 Department of Hematology/Oncology, University of Münster, Münster, Germany; 11 Hematology Department, University Hospital La Fe, Valencia, Spain; 12 Deutsche Klinik für Diagnostik, KMT Zentrum, Wiesbaden, Germany; 13 Department of Hematology, Azienda Ospedaliera Universitaria Careggi, Firenze, Italy; 14 Bone Marrow Transplantation Centre, University Hospital Eppendorf, Hamburg, Germany; 15 Department of Haematology II, Ospedale San Martino, Genova, Italy; 16 Department of Hematology, Hemostasis, Oncology, and Stem Cell Transplantation, Hannover Medical School, Hannover, Germany; 17 CHU Bordeaux, Service d’hematologie et therapie cellulaire, F-33000 Bordeaux, France; 18 Division of Hematology and Bone Marrow Transplantation, The Chaim Sheba Medical Center, Tel-Hashomer, Ramat-Gan, Israel

## Abstract

The role of umbilical cord blood transplantation (CBT) in acute myeloid leukemia (AML) patients with active disease at allogeneic hematopoietic cell transplantation (allo-HCT) remains poorly investigated. In this study, we compared transplantation outcomes of 2963 patients with primary refractory or relapsed AML given CBT, 10/10 HLA-matched UD, or 9/10 HLA-matched UD allo-HCT from 2004 to 2015 at EBMT-affiliated centers. Neutrophil engraftment and complete remission rates in CBT, UD 10/10, and UD 9/10 recipients were 75 and 48%, 93 and 69%, and 93 and 70%, respectively. In multivariate Cox analyses, in comparison with CBT (*n* = 285), UD 10/10 recipients (*n* = 2001) had a lower incidence of relapse (HR = 0.7, *P* = 0.001), a lower incidence of non relapse mortality (HR = 0.6, *P* < 0.001), better GVHD-free and leukemia-free survival (GRFS, HR = 0.8, *P* < 0.001) and better survival (HR = 0.6, *P* < 0.001). Further, in comparison with CBT, 9/10 UD recipients (*n* = 677) also had a lower incidence of relapse (HR = 0.8, *P* = 0.02), a lower incidence of nonrelapse mortality (HR = 0.7, *P* = 0.008), better GRFS (HR = 0.8, *P* = 0.01) and better survival (HR = 0.7, *P* < 0.001). In summary, these data suggest that in AML patients with active disease at transplantation, allo-HCT with UD results in better transplantation outcomes than CBT.

## Introduction

Allogeneic hematopoietic stem cell transplantation (allo-HCT) has remained the only potentially curative option for most patients with relapsed or primary refractory acute myeloid leukemia (AML)^1,2^. This approach is thought to rely mostly on graft-versus-leukemia (GvL) effects for tumor eradication^3–5^.

For adult patients with AML in complete remission (CR) who lack a suitable human leukocyte antigen (HLA)-identical sibling, and cord blood transplantation (CBT) has proved to be an adequate alternative to HLA-matched unrelated (UD) bone marrow (BM)/peripheral blood stem cell (PBSC) transplantation^6–8^.

We recently compared GvL effects following low-intensity non myeloablative conditioning regimen according to donor type^9^. We observed that, in comparison with patients given grafts from HLA-matched unrelated donor (UD 10/10), those receiving CBT had similar overall survival (OS) but better GVHD-free and relapse-free survival (GRFS). These results are in line with another study from the Fred Hutchinson Cancer Research Center that demonstrated that, among AML patients in CR but with minimal residual disease at transplantation, those receiving CBT had at least as good overall survival (OS) than those given grafts from UD10/10, and better OS than those receiving grafts from HLA-mismatched UD^10^.

We hypothesized that, since CBT provides better outcome than UD in AML patients with minimal residual disease at transplantation, transplantation outcomes might be better with CBT than with UD in AML patients with active disease at transplantation. In order to challenge this hypothesis, we performed a large registry study comparing CBT with UD 10/10 or 1-antigen HLA-mismatched UD (UD 9/10) in patients with primary refractory or untreated/refractory relapsed AML.

## Patients and Methods

### Data collection

This is a retrospective, multicenter registry-based study performed by the Acute Leukemia Working Party (ALWP) of the European society for Blood and Marrow Transplantation (EBMT) and by Eurocord. EBMT registry is a voluntary working group of more than 500 transplant centers, participants of which are required once a year to report all consecutive stem cell transplantations and follow-up. Audits are routinely performed to determine the accuracy of the data. Eurocord collects data on CBT performed in >50 countries worldwide and >500 transplant centers, mainly EBMT centers.

Inclusion criteria were adult (≥18 years) patients, de novo or secondary AML, primary refractory (defined as absence of CR (<5% marrow blasts) achievement after induction chemotherapy^1^) or in first or second relapse at transplantation, transplantation between 2004 and 2015, and either a 10/10 HLA-matched unrelated donor (UD 10/10), a 9/10 HLA-matched unrelated donor (UD 9/10), or a single or double CBT. For UD, HLA-A, HLA-B, HLA-C, HLA-DR, and HLA-DQ were typed at the allelic level. For CBT, HLA-compatibility requirements followed the current practice of antigen level typing for HLA-A and HLA-B and allele level typing of HLA-DRB1. CB units were 4–6/6 HLA-A, HLA-B, and HLA-DRB1 matched to the recipient and to the other unit in case of double CBT in most patients^11,12^. HLA disparities between each unit and the recipient and between the two units were not necessarily at the same loci. Grading of acute and chronic GVHD was performed using established criteria^13^.

For the purpose of this study, all necessary data were collected according to EBMT guidelines.

### Ethics approval and consent to participate

The scientific boards of the ALWP of EBMT and of Eurocord approved this study. Since 1990, patients have provided informed consent authorizing the use of their personal information for research purposes.

### Statistical analyses

Data from all patients meeting the inclusion/exclusion criteria were included in the analyses. Start time was date of transplant for all endpoints. Neutrophil engraftment was defined as first of 3 consecutive days with a neutrophil count of at least 0.5 × 10^9^/L.

Cumulative incidence functions were used for relapse incidence and non relapse mortality (NRM) in a competing risk setting, since death and relapse were competing together. For estimating the cumulative incidence of engraftment and chronic GVHD, death was considered as a competing event. Overall (OS) and leukemia-free (LFS) survivals were estimated using the Kaplan-Meier estimates. GVHD and relapse-free survival (GRFS) was defined as being alive with neither grade III-IV acute GVHD, extensive chronic GVHD nor disease relapse^14^.

The main characteristics at diagnosis and at transplantation were compared between CBT and 10/10 or 9/10 UD groups using Kruskall Walis tests for quantitative variables, Chi-square test or Fisher exact test for categorical variables. Univariate analyses were done using Gray’s test for cumulative incidence function and log rank test for OS and LFS.

Associations between donor type and transplantation outcomes were evaluated in multivariable analyses, using Cox proportional hazards. We used propensity scores (PS) matching to control for pre-treatment imbalances on observed variables. The following factors were included in the propensity score model: age, year of transplant, status at transplantation (primary refractory or relapse), diagnosis (de novo or secondary AML), sex matching (female to male vs other), patient and donor CMV serology, conditioning intensity (RIC or MAC). The estimation of propensity score was performed using generalized boosted models^15^. We weighted the 3 groups receiving either CBT, 10/10, or 9/10 UD by estimating the Average Treatment Effect (ATE). We checked the balance between the groups looking to ATE Weighted means. Then, we used pairwise ATEs to fit weighted KM and Cox models. All tests were two sided. The type I error rate was fixed at 0.05 for determination of factors associated with time to event outcomes. Analyses were performed using the R statistical software version 3.4.0. Propensity score analysis was performed using the mnps function of the Twang package (http://cran.rproject.org/web/packages/twang/vignettes/twang.pdf), and weighted analyses using the survey package.

## Results

### Patients and donors

Data from 2963 patients with primary or secondary AML were included in this study (Table 1). Two hundred eighty five patients underwent a single (*n* = 175) or double (*n* = 110) CBT, 2001 an UD 10/10 and 677 an UD 9/10 allo-HCT. In comparison to 10/10 UD or 9/10 UD recipients, CBT patients were younger (48 vs. 55 and 55 years, respectively, *P* < 0.001), were less frequently transplanted with primary refractory disease (37% vs. 50 and 42%, respectively, *P* = 0.009), and were more frequently conditioned with a myeloablative regimen (52% vs. 46 and 42%, respectively, *P* < 0.001). However, they received less frequently in vivo T cell depleting agents such as ATG or alemtuzumab (62% vs. 75 and 85%, respectively, *P* < 0.0001).

**Table 1:** Patient and transplant characteristics

	CBT (*n* = 285)	UD 10/10 (*n* = 2001)	UD 9/10 (*n* = 677)	*P* value^a^
Median patient age, year (range)	47 (18–71)	55 (18–77)	55 (18–77)	<0.001
Median follow-up (pts alive), month (range)	23 (2–121)	22 (1–146)	27 (1–136)	<0.001
Median year of transplantation	2009	2011	2011	<0.001
Recipient gender M, # (%)	139 (49)	1090 (55)	358 (53)	0.2
F donor to M recipient, # (%)	61 (24)	226 (12)	113 (17)	<0.001
Karnofsky performance status at Tx				
<80	37 (15)	268 (14)	86 (13)	0.8
>=80	216 (85)	1611 (86)	555 (87)	
Missing	32	122	36	
Diagnosis, # (%)				
De novo AML	204 (72)	1282 (64)	435 (64)	0.04
Secondary AML	81 (28)	719 (36)	242 (36)	
Status at transplantation, # (%)				
Primary refractory	106 (37)	999 (50)	286 (42)	<0.001
First relapse	146 (50)	876 (44)	336 (50)	
Second relapse	37 (13)	126 (6)	55 (8)	
Cytogenetics, # (%)				
Good risk^b^	8 (4)	56 (5)	23 (5)	0.001
Intermediate risk^c^	44 (25)	178 (15)	75 (18)	
High risk^d^	46 (26)	201 (17)	85 (20)	
Secondary AML	81 (45)	719 (62)	242 (57)	
Not reported/failed	106	847	252	
FLT3-ITD, # (%)				
Negative	28 (58)	150 (61)	52 (52)	0.3
Positive	20 (42)	94 (39)	48 (48)	
Missing	237	1757	577	
Stem cell source				
Bone marrow	152 (8)	56 (8)		
Peripheral blood stem cells	1849 (92)	620 (92)		
Single CBT			175 (61)	
Double CBT			110 (39)	
Patient CMV seropositive, # (%)	169 (69)	1222 (63)	430 (66)	0.1
Conditioning intensity, # (%)				
Myeloablative (MAC)	148 (52)	901 (46)	276 (42)	0.009
Reduced-intensity (RIC)	135 (48)	1062 (54)	387 (58)	
Conditioning regimen, # (%)				
Cy-TBI	106 (39)	208 (11)	70 (11)	<0.001
Flu-TBI	10 (4)	204 (10)	44 (7)	
BuCy	13 (5)	207 (11)	60 (9)	
BuFlu	6 (2)	323 (17)	98 (15)	
FluMel	15 (5)	255 (13)	98 (15)	
TBF	66 (24)	32 (2)	24 (4)	
Flamsa TBI/chemo	23 (8)	454 (23)	184 (28)	
Other	35 (13)	273 (14)	83 (12)	
Missing	11	45	16	
In vivo T-cell depletion, # (%)				
Yes	98 (38)	485 (25)	101 (15)	<0.001
No	159 (62)	1491 (75)	569 (85)	
Missing	28	25	7	
Postgrafting immunosuppression, # (%)				
CSP alone	58 (23)	206 (11)	64 (10)	<0.001
CSP (or tacro) + MTX +/− MMF	13 (5)	686 (35)	220 (33)	
CSP (or tacro) + MMF	174 (69)	966 (50)	345 (52)	
Post-transplant cyclophosphamide	7 (3)	28 (1)	13 (2)	
Other	1 (0)	61 (3)	24 (4)	
Missing	32	54	11	

*M* male, *CR* complete remission, # number of patients, *UD* unrelated donor, *CBT* cord blood transplantation, *CSP* cyclosporine, *MMF* mycophenolate mofetil, *FLT3-ITD* FMS-related tyrosine kinase 3 internal tandem duplication, *Cy* cyclophosphamide, *TBI* total body irradiation, *Bu* busulfan, *Flu* fludarabine, *Mel* Melphalan, *TBF* thiotepa + busulfan + fludarabine, Flamsa fludarabine + amsacrine + cytarabine, *MTX* methotrexate

^a^calculated with *χ*^b^ statistics for categorical variables and Mann-Whitney test for continuous variables

^b^defined as t(8;21), t(15;17), inv or del (16), or acute promyelocyticleukemia, these abnormalities only or combined with others

^c^defined as all cytogenetics not belonging to the good or high risk (including trisomias)

^d^defined as 11q23 abnormalities, complex caryotype, abnormalities of chromosomes 5 and 7

### Engraftment and GVHD

Cumulative incidence of neutrophil engraftment at day 60 was 73%, 94 and 93% in CBT, UD 10/10 and UD 9/10, respectively (*P* < 0.001). Cumulative incidence of grades II-IV acute GVHD were 27%, 30 and 36% in CBT, UD10/10, and UD 9/10 recipients, respectively (*P* = 0.002). For grade III-IV acute GVHD, the figures were 12%, 13 and 17%, respectively (*P* = 0.02). Further, the 2-year cumulative incidences of chronic and extensive chronic GVHD were lower in CBT patients (16 and 6%, respectively) than in UD 10/10 (28 and 12%, respectively) or UD 9/10 patients (29 and 14%, respectively) (*P* < 0.001 and *P* = 0.004, respectively).

### CR achievement, relapse and NRM

**Fig. 1:**
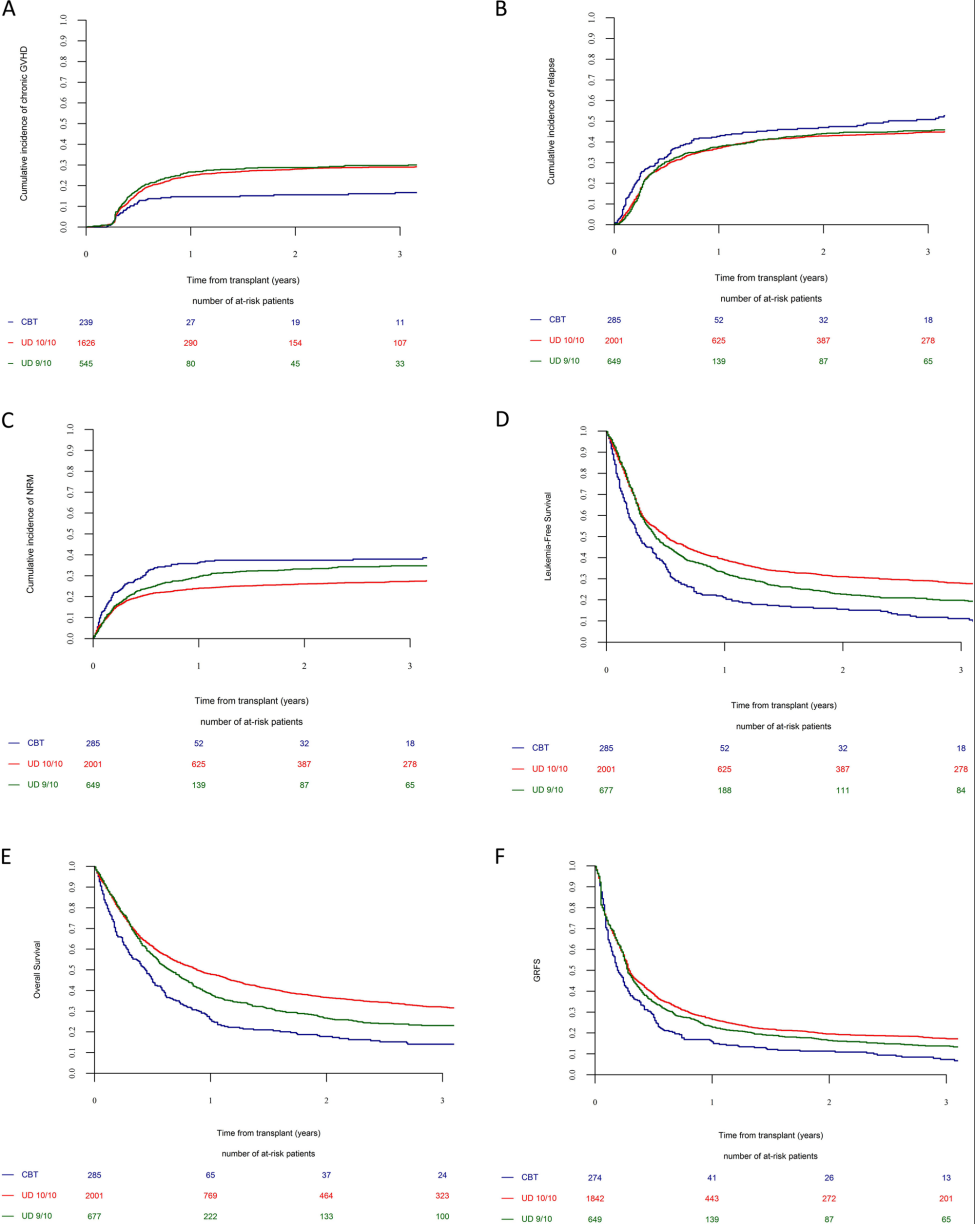
Impact of donor type on transplantation outcomes. **a** Chronic GVHD. **b** Relapse. **c** Non relapse mortality. **d** LFS. **e** OS. **f** GRFS

Following transplantation, 69% of UD 10/10 recipients, 70% of 9/10 recipients versus 48% of CBT recipients achieved a CR within 100 days (*P* < 0.001). Two-year incidences of relapse and NRM were 43 and 26% in UD 10/10 recipients, 44 and 33% in UD 9/10 recipients and 47 and 38% in CBT recipients, respectively (Fig. 1). In multivariate Cox analyses, in comparison to CBT patients, UD 10/10 recipients had a lower incidence of relapse (HR = 0.7, *P* = 0.001) and a lower incidence of NRM (HR = 0.6, *P* < 0.001). Further, in comparison with CBT patients UD 9/10 recipients had also a lower incidence of relapse (HR = 0.8, *P* = 0.02) and a lower incidence of NRM (HR = 0.7, *P* = 0.008).

Other factors associated with the relapse incidence in multivariate analysis included secondary versus primary AML (HR = 0.8, *P* = 0.01), relapsed versus primary refractory AML (HR = 1.2, *P* = 0.02) and older age at transplantation (HR per ten year = 0.9, *P* < 0.001). Further, other factors associated with NRM in multivariate analyses included older age (HR = 1.1, *P* < 0.001), female donor to male recipient (HR = 1.2, *P* = 0.05) and patient CMV seropositivity (HR = 1.3, *P* = 0.002).

### OS, LFS, GRFS

At 2-year, OS, LFS, and GRFS were 37, 31, and 20% in UD 10/10 recipients, 27, 23, and 16% in UD 9/10 recipients, and 18, 16 and 11% in CBT patients, respectively. In multivariate Cox analyses, in comparison with CBT, UD 10/10 recipients had a better LFS (HR = 0.6, *P* < 0.001), a better GRFS (HR = 0.8, *P* < 0.001) and a better OS (HR = 0.6, *P* < 0.001) (Table 2). Further, in comparison to CBT patients, UD 9/10 recipients had also a better LFS (HR = 0.7, *P* < 0.001), a better GRFS (HR = 0.8, *P* = 0.01) and a better OS (HR = 0.7, *P* < 0.001) (Table 2).

**Table 2:** Impact of donor types on transplantation outcomes in multivariate Cox models

	HR	95% CI	*P*
Relapse			
CBT	1	–	–
UD 10/10	0.7	0.6–0.9	<0.001
UD 9/10	0.8	0.6–1.0	0.02
Non relapse mortality			
CBT	1	–	–
UD 10/10	0.6	0.4–0.7	<0.001
UD 9/10	0.7	0.5–0.9	0.007
Leukemia-free survival			
CBT	1	–	–
UD 10/10	0.6	0.6–0.8	<0.001
UD 9/10	0.7	0.6–0.9	<0.001
Overall survival			
CBT	1	–	–
UD 10/10	0.6	0.5–0.7	<0.001
UD 9/10	0.7	0.6–0.9	0.001
GVHD-free and relapse-free survival			
CBT	1	–	–
UD 10/10	0.8	0.7–0.9	<0.001
UD 9/10	0.8	0.7–1.0	0.01

Other factors associated with transplantation outcomes in multivariate analyses included relapsed versus primary refractory AML that predicted poor LFS (HR = 1.1, *P* = 0.02) and OS (HR = 1.1, *P* = 0.04), while patient CMV seropositivity predicted for poor LFS (HR = 1.2, *P* = 0.003), OS (HR = 1.2, *P* = 0.003), and GRFS (HR = 1.1, *P* = 0.01).

Among CBT recipients, 38% of patients died because of the original disease, 24% because of an infection, and 5% because of GVHD. Among UD 10/10 recipients the figures were 31, 13, and 7% respectively. Finally, among 9/10 recipients, the figures were 34, 15, and 13%, respectively.

### Propensity score analysis

Given the differences in the study population between the 3 groups we also performed analyses weighted with propensity score. These analyses showed that in comparison with CBT recipients, UD 10/10 patients had lower incidences of relapse and of nonrelapse mortality translating to better LFS, OS, and GRFS (Table 3). Further, in comparison with CBT recipients, UD 9/10 patients had a lower incidence of relapse, a not significantly different incidence of NRM, as well as better LFS, OS, and GRFS (Table 3).

**Table 3:** Impact of donor types on transplantation outcomes using Cox models weighted on propensity score

	HR	95% CI	*P*
Relapse			
CBT	1	–	–
UD 10/10	0.7	0.5–0.8	<0.001
UD 9/10	0.87	0.5–0.9	0.005
Non relapse mortality			
CBT	1	–	–
UD 10/10	0.6	0.5–0.8	<0.001
UD 9/10	0.8	0.6–1.1	0.1
Leukemia-free survival			
CBT	1	–	–
UD 10/10	0.6	0.5–0.8	<0.001
UD 9/10	0.7	0.6–0.9	0.002
Overall survival			
CBT	1	–	–
UD 10/10	0.6	0.5–0.8	<0.001
UD 9/10	0.8	0.6–0.9	0.004
GVHD-free and relapse-free survival			
CBT	1	–	–
UD 10/10	0.7	0.6–0.9	<0.001
UD 9/10	0.8	0.6–0.9	0.008

## Discussion

Based on recent data demonstrating that in patients with MRD at transplantation, CBT did as least as good as UD transplantation, we performed a survey comparing the outcomes of patients with refractory/relapsed AML transplanted with a UD vs. a CB; several observations were made.

First, we observed a high incidence of primary graft failure, as well as a low incidence of CR achievement in CBT recipients. The high incidence of graft failure in CBT recipient was due in a large part to early AML progression precluding neutrophil engraftment since only 49% of CBT recipients achieved a CR after transplantation. This suggests that the kinetic of GvL effects is slower in CBT than in UD recipients. This might be due to the lower number of T cells infused with CBT, as well as to their mostly immature status^16^.

Second, we observed a significantly higher NRM in CBT patients than in UD recipients. While this could probably be attributed in a part to the slow neutrophil engraftment associated with CBT, it could also be due to delayed immune reconstitution in CBT patients since the higher non relapse mortality in CBT than in UD recipients was due to infections. One cannot include that the large use of ATG among CBT recipients was in part the cause of this high infection-related mortality among CBT recipients^17^. Recent advances in the field of CBT engineering are likely to improve the safety of CBT in AML patients with active disease at transplantation^18–20^.

Consequently to the high disease-related and infection-related mortality among CBT recipients, OS, LFS, and GRFS were significantly better both in UD 10/10 and in UD 9/10 recipients than in CBT patients. These results are in contrast with those observed in AML patients in CR with or without MRD, where CBT did at least as good as UD allo-HCT^8–10^.

There are some limitations in our study. They include the heterogeneity in patients characteristics between the different groups and the lack of relevant data in the database such as blast counts at transplantation, comorbidity score, or donor T cell reconstitution after allo-HCT. We tried to address the heterogeneity question by comparing transplantation outcomes between the three different groups by using both multivariate Cox models and propensity weighted Cox models.

In summary, these data suggest that in AML patients with active disease at transplantation, allo-HCT with UD results in better transplantation outcomes than CBT.

## List of institutions

The EBMT registry is a voluntary working group of more than 500 transplant centers, participants of which are required once a year to report all consecutive stem cell transplantations and follow-up. The list of institutions reporting data included in this study is provided in the supplemental data.
